# Epidemiological features of hypermucoviscous *Klebsiella pneumoniae* infection in a coastal city of China: clinical and molecular perspectives

**DOI:** 10.3389/fcimb.2026.1732291

**Published:** 2026-02-09

**Authors:** Fengzhen Yang, Lingyan Hou, JinCheng Rong, Xiaohui Chi, Qi Zhao, Maoli Yi

**Affiliations:** 1 Yantai Yuhuangding Hospital, Yantai, China; 2 Laizhou People’s Hospital, Yantai, China

**Keywords:** genome sequencing, hypermucoviscous, hypervirulent, *Klebsiella pneumoniae*, virulence determinant

## Abstract

**Purpose:**

The present study aimed to investigate the overall clinical and molecular characteristics of hypermucoviscous *Klebsiella pneumoniae* (hmKP) infection in Yantai, China.

**Methods:**

A retrospective study was conducted in Yantai Yuhuangding Hospital from October 2024 to December 2024. As the inclusion criterion, all clinical isolates of *K. pneumoniae* were analyzed by a string test, in which an inoculation loop was used to generate sticky strings of >5 mm length from a *K. pneumoniae* colony. The isolates showing a positive test result were designated as hmKP. The clinical characteristics of patients with hmKP infection were retrospectively reviewed. Antibiotic susceptibility tests were performed for all hmKP isolates, and whole-genome sequencing studies were conducted for these isolates to determine their epidemiological characteristics.

**Results:**

A total of 88 unique hmKP isolates were collected from 485 strains of *K. pneumoniae*, accounting for 18.1%. Patients with hmKP infection were distributed across different age groups, clinical departments, and specimen types. Whole-genome sequencing revealed the predominance of K1/ST23 and K2/ST65 clones in the hmKP group. Virulence determinants such as *rmpA*, *rmpA2*, *iuc*, *iro*, *peg-344*, and *clb* were commonly present in the hmKP isolates. All hmKP isolates carried multiple resistance genes; however, the antibiotic resistance phenotype did not fully match with the resistance genes. Additionally, two hypervirulent carbapenem-resistant *Klebsiella pneumoniae*(HCKP) strains were detected in this study.

**Conclusion:**

HmKP infection is prevalent in Yantai, China, with dominant K1/ST23 and K2/ST65 clones carrying prevalent virulence genes. Vigilance is required to prevent the spread of HCKP in clinical practice.

## Introduction

*Klebsiella pneumoniae*, which was first reported in 1882, is an important opportunistic pathogen causing various infectious diseases, including pneumonia, urinary tract infections, bacteremia, and liver abscesses (Hu et al., 2020; Xu et al., 2021). A key characteristic of *K. pneumoniae* is its ability to acquire new genetic materials (Namikawa et al., 2023). Based on virulence gene carriage, two distinct pathotypes - classical *K. pneumoniae* and hypervirulent *K. pneumoniae* (hvKP), are currently spreading worldwide, with each type posing a unique challenge to clinicians (Martin, 2018). Although hvKP has attracted considerable interest in the clinical field, its diagnosis relies on sequencing technology or polymerase chain reaction (PCR), which are seldom available in conventional laboratories. In contrast, a string test, in which an inoculation loop is used to generate sticky strings from a *K. pneumoniae* colony, is a simple procedure that can be easily performed in conventional laboratories, and it can be utilized to classify *K. pneumoniae* strains into hypermucoviscous *K. pneumoniae* (hmKP, positive result in string test) and non-hypermucoviscous *K. pneumoniae* (non-hmKP, negative result in string test) (Jiang et al., 2022; Wang et al., 2022). Although studies have suggested that hvKP strains often exhibit a hypermucoviscous phenotype (i.e., hmKP) (Martin, 2018; Zhang S, Zhang et al., 2019), the two are not entirely synonymous. The diagnosis of hvKP is based on the presence of virulence genes such as *rmpA*, *iuc*, or *peg-344*, while hmKP is defined phenotypically by the string test.

The present study aimed to systematically investigate the overall clinical and molecular characteristics of hmKP infection in Yantai, China, from the following perspectives: (1) easy implementation of the diagnostic test for hmKP in conventional laboratories, (2) rapid and more efficient detection of hmKP infection, and (3) improvement of patient prognosis through early diagnosis and clinical treatment.

## Materials and methods

### Bacterial isolate collection

This retrospective study was conducted in Yantai Yuhuangding Hospital, a 3000-bed tertiary teaching hospital in Yantai, China, from October to December 2024. As the inclusion criterion, all clinical isolates of *K. pneumoniae* were subjected to a string test, in which an inoculation loop was used to generate sticky strings of >5 mm length from a *K. pneumoniae* colony. The isolates showing a positive result in the string test were designated as hmKP. All selected hmKP isolates were cultured in blood agar at 35 °C for 20–24 h and then stored in skimmed milk at -80 °C until use. Duplicate isolates collected from the same patient within 3 months were excluded.

### Clinical data collection

The clinical data of all study participants were reviewed retrospectively through medical records, including age, gender, clinical department for treatment, basic diseases (hypertension, diabetes, malignant tumor, etc.), laboratory inflammatory indicators (white blood cell count, C-reactive protein (CRP), and procalcitonin (PCT)), hospital stay, and clinical outcome.

### Strain identification and antibiotic susceptibility tests

All the collected hmKP isolates were removed from the -80 °C refrigerator, transferred to blood agar plates, and incubated at 35 °C for 20–24 h. Strain identification was performed through the matrix-assisted laser desorption/ionization time-of-flight mass spectrometry (Bruker Daltonics, Karlsruhe, Germany). Antibiotic susceptibility tests were conducted on the VITEK^®^2 compact system (Biom´erieux, Marcy l’Etoile, France) with the following antibiotics: cefazolin, cefuroxime, cefotetan, ceftazidime, ceftriaxone, cefepime, aztreonam, ampicillin/sulbactam, amoxicillin/clavulanic acid, piperacillin/tazobactam, cefoperazone/sulbactam, meropenem, imipenem, ciprofloxacin, levofloxacin, gentamicin, tobramycin, amikacin, and trimethoprim/sulfamethoxazole. All procedures were conducted in accordance with the manufacturer’s instructions. *Escherichia coli* ATCC 25922, *E. coli* ATCC 35218, and *Pseudomonas aeruginosa* ATCC 27853 were used as quality control strains. The minimum inhibitory concentration breakpoints were interpreted according to the CLSI M100-S34 standard.

### Genome sequencing

A Genomic DNA Kit (Tiangen, Beijing, China; DP305) was used to extract the genomic DNA. The DNA concentration was quantified and verified by a NanoDrop™ 2000 spectrophotometer (Thermo Fisher Scientific, Waltham, MA, USA) and agarose gel electrophoresis, respectively. At least 50 ng of extracted DNA was required for library preparation. The TruePrep™ DNA Library Prep Kit V2 (Vazyme, Jiangsu, China) was used for library preparation. By using a single “transposase” enzymatic reaction, sample DNA was simultaneously fragmented and tagged with adapters. An optimized, limited-cycle PCR protocol was used to amplify tagged DNA, and sequencing indexes were added. Individual libraries were evaluated on a QIAxcel Advanced Automatic Nucleic Acid Analyzer and then quantified by quantitative real-time PCR (qPCR) using the KAPA SYBR^®^ FAST qPCR Kit. Finally, the library was sequenced on an Illumina HiSeq 2500 sequencing platform (Illumina Inc., San Diego, CA, USA), and 150 bp paired-end reads were generated. Low-quality reads were removed from the raw data, and the clean data were assembled through SPAdes v3.13.

Phylogenetic analysis was performed based on single-nucleotide polymorphism (SNP) loci from seven core genes, including *phoE*, *gapA*, *mdh*, *tonB*, *pgi*, *rpoB*, and *infB*. The phylogenetic tree was constructed using MEGA 11 with 1,000 bootstrap replicates. Sequence typing was conducted by multilocus sequence typing (https://cge.food.dtu.dk/services/MLST/), and the capsular serotype was determined by comparison with Kaptive (https://pathogen.watch/). Virulence genes and antimicrobial resistance genes were identified by BLAST using VirulenceFinder (https://cge.food.dtu.dk/services/VirulenceFinder/) and ResFinder (https://cge.food.dtu.dk/services/ResFinder/), respectively, with thresholds of 90% identity and a minimum length coverage of 80%. In this study, hvKP strains were identified based on the presence of the *peg-344* and *iuc* genes. Hypervirulent carbapenem-resistant *Klebsiella pneumoniae* (HCKP) was defined as hvKP strains that were also resistant to carbapenems.

### Statistical analyses

SPSS Statistics 26 software (IBM Corporation, NY, USA) was used for data management and analysis. Categorical data were summarized using numbers and percentages. Continuous variables were represented as means ± standard deviations. Fisher’s exact test was performed for comparison of categorical variables. Odds ratios (ORs) and their corresponding 95% confidence intervals (CIs) were calculated to quantify the strength of associations. A two-sided p-value of less than 0.05 was considered statistically significant.

## Results

### Baseline characteristics of the patients

According to the inclusion criterion, 88 unique hmKP isolates were collected from 485 strains of *K. pneumoniae*, accounting for 18.1%. The baseline characteristics of hmKP infected patients are listed in **Table 1**. The isolates were primarily derived from pus, sputum, and blood samples and to a lesser extent from tissues, bile, and cerebrospinal fluid. The age of the patients ranged from 21 to 92 years (average age: 62.56 ± 13.87 years). Patients were distributed in almost all clinical departments of the hospital, with most patients admitted to the intensive care unit (ICU) and hepatobiliary department. Hypertension, diabetes, and liver abscess were the most common underlying diseases in hmKP infection. Laboratory tests revealed that patients with hmKp infection exhibited elevated inflammatory markers and prolonged hospital stays. Furthermore, over 20% of patients had a poor prognosis.

**Table 1:** Baseline characteristics of patients with hmKP infection.

Variates	Results
Demographics	
Male	52 (59.1%)
Female	36 (40.9%)
Age(15-65y)	49 (55.7%)
Age(>66y)	39 (44.3%)
Main departments	
Intensive care unit	22 (25.0%)
Hepatobiliary department	22 (25.0%)
Main specimen types	
Pus	35 (39.8%)
Sputum	23 (26.1%)
Blood	15 (17.0%)
Underlying conditions	
Hypertension	37 (42.0%)
Diabetes	35 (39.8%)
Liver abscess	21 (23.9%)
Malignancy	12 (13.6%)
Laboratory findings	
White blood cell (*10^9^/L)	10.96 ± 5.84
CRP (mg/L)	144.91 ± 103.09
PCT (ng/ml)	14.50 ± 27.69
Hospital stay and prognosis	
Hospital stay	17.85 ± 21.64
Improved	69 (78.4%)
Aggravated	11 (12.5%)
Died	8 (9.1%)

### Phylogenetic analysis

**Figure 1:**
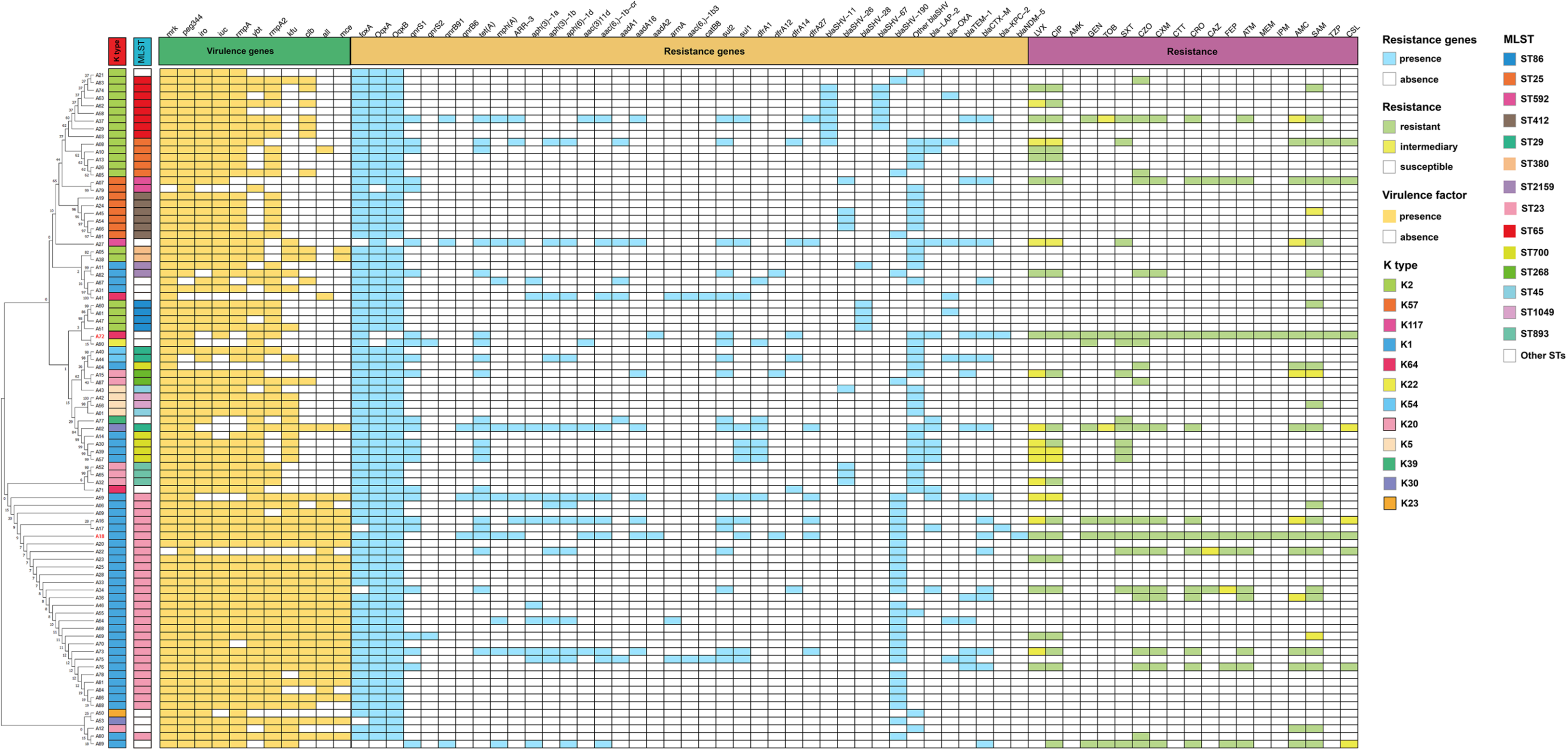
Molecular epidemiological characteristics of the 88 strains of hmKP. LVX, levofloxacin; CIP, ciprofloxacin; AMK, amikacin; GEN, gentamicin; TOB, tobramycin; SXT, trimethoprim/sulfamethoxazole; CZO, cefazolin; CXM, cefuroxime; CTT, cefotetan; CRO, ceftriaxone; CAZ, ceftazidime; FEP, cefepime; ATM, aztreonam; MEM, meropenem; IPM, imipenem; AMC, amoxicillin/clavulanic acid; SAM, ampicillin/sulbactam; TZP, piperacillin/tazobactam; CSL, cefoperazone/sulbactam.

The phylogenetic tree constructed from SNP loci (**Figure 1**) revealed a dispersed evolutionary pattern among the isolates, and strains that were closely related on the tree (e.g., A21, A83, A74 and A63) were distributed across different departments.

### Capsular serotype and sequence type

The capsule serotype (K) and sequence type (ST) of the hmKP isolates were relatively dispersed (**Figure 1**). A total of 12 capsule serotypes and 27 sequence types were detected in the 88 hmKP strains. Among these, K1 was the most predominant capsular serotype, accounting for 44.3% of the hmKP strains (n = 39), followed by K2 (22.7%, n = 20). ST23 was the major sequence type, accounting for 32.9% of the hmKP strains (n = 29), followed by ST65 (9.1%, n = 8). Correlation analysis between capsule serotypes and sequence types was performed (**Table 2**). The most remarkable finding was an exceptionally strong, near-exclusive association between K54 and ST29 (OR = 865.00, 95% CI: 14.10–53,074.81, *p* < 0.001). Other notable pairings, including K57/ST412 (OR = 418.60) and K20/ST893 (OR = 165.00), also showed high statistical significance(*p* < 0.001). Furthermore, this study confirmed the presence of globally recognized hypervirulent clones in our collection, most notably the classical K1/ST23 (OR = 278.14) and K2/ST65 (OR = 93.16) lineages. Additional associations, such as K5 with ST45/ST1049 and K57 with ST592, were also statistically significant, though their effect estimates were less precise, as indicated by wider confidence intervals. Conversely, several pairings, including K1 with ST2159, ST449, ST367, and ST5214, showed no statistically significant association (*p*>0.05), indicating these STs are not characteristically linked to the K1 serotype.

**Table 2:** Correlation analysis between capsule serotypes and sequence types.

Capsule serotypes/sequence types	*p* values	Odds ratios	95%confidence intervals
K2/ST65	0.0001	93.16	5.05-1719.09
K1/ST23	0.0001	278.14	15.71-4922.72
K57/ST412	0.0001	418.60	18.12-9669.70
K20/ST893	0.0002	165.00	7.07-3851.96
K54/ST29	0.0003	865.00	14.10-53074.81
K2/ST25	0.0004	48.61	2.55-926.14
K5/ST45 K5/ST1049	0.0016	169.00	6.32-4521.19
K2/ST86	0.0021	37.36	1.92-728.89
K20/ST268	0.0039	91.67	3.81-2206.95
K57/ST592	0.0073	61.92	2.68-1430.43
K117/ST828 K22/ST34 K23/ST39 K39/ST514	0.0114	525.00	7.57-36412.36
K1/ST700	0.0147	15.78	0.84-294.85
K30/ST29 K30/ST1027	0.0227	173.00	4.80-6235.07
K64/ST11 K64/ST1647 K64/ST505	0.0341	102.60	3.29-3196.17
K2/ST380	0.0496	18.51	0.85-402.64
K20/ST420	0.0682	45.00	1.64-1238.52
K1/ST2159	0.1936	6.6	0.31-141.60
K2/ST375	0.2273	10.54	0.41-268.07
K1/ST449 K1/ST367 K1/ST5214	0.4432	3.85	0.15-97.34

### Virulence determinants

The virulence determinants of the 88 hmKP isolates are listed in **Figure 1**. The following virulence determinants were prevalent in the 88 hmKP strains: *mrk* (97.7%, n = 86), *peg-344* (95.5%, n = 84), *iro* (89.8%, n = 79), *iuc* (87.5%, n =77), *rmpA* (86.4%, n = 76), *ybt* (75.0%, n = 66), *rmpA2* (75.0%, n = 66), *kfu* (53.4%, n = 47), *clb* (44.3%, n = 39), *all* (36.4%, n = 32), and *mce* (33.0%, n = 29). The average number of virulence determinants carried by each hmKP strain was 7.74.

### Resistance gene and antibiotic susceptibility tests

The antimicrobial resistance genes and antibiotic sensitivity of all hmKP strains were detected, and the results are shown in **Figure 1**. The MIC and kb values of all antimicrobials tested for each hmKP isolate are provided in **Supplementary Table 1**. All strains carried β-lactam resistance genes, while 97.7% and 94.3% of the strains carried quinolone and fosfomycin resistance genes, respectively; the distribution of other resistance genes was scattered. A noteworthy finding is that, of the 88 hmKP strains, two strains exhibited *bla*_KPC_ resistance genes, and one strain carried the *bla*_NDM_ resistance gene. Antibiotic sensitivity tests revealed that most hmKP strains were sensitive to the tested antibiotics. Except for cefazolin, ampicillin/sulbactam, and ciprofloxacin with resistance rates greater than 20%, the resistance rates for other antibiotics were below 20%. Additionally, two HCKP strains(A72, A18) were detected, as highlighted in red in **Figure 1**.

## Discussion

In recent years, the incidence of *K. pneumoniae* infection has drastically increased (Zhang S, Zhang et al., 2019; Walker, 2020; Zhang et al., 2020; Nakamura-Silva et al., 2022; Le et al., 2023). In many global regions, *K. pneumoniae* has become the prevalent pathogen with the highest isolation rate (Zhang S, Zhang et al., 2019; Gan et al., 2020; Spadar et al., 2021). Because of its high isolation rate and strong pathogenicity, *K. pneumoniae*, particularly hvKP strains, has increasingly attracted the attention of researchers worldwide (Marr, 2019; de Oliveira Franco et al., 2024). The hvKP strain is highly pathogenic, with a high mortality rate and a high risk of dispersal. However, the diagnosis of hvKP strains relies on sequencing or PCR technology, which is expensive, time-consuming, and difficult to implement in conventional laboratories (Russo et al., 2018). In contrast, the diagnostic procedure for hmKP and non-hmKP strains is rapid, simple, and economical (Dentice Maidana et al., 2023; Jin et al., 2023; Campanero-Rhodes et al., 2024). Moreover, it could aid clinical practice and therefore has a high practical value.

Traditionally, hmKP infection has been predominantly associated with liver abscesses (Choby et al., 2020; Valente-Acosta et al., 2022). In the present study, however, we found hmKP to be highly prevalent, accounting for 18.1% of all *K. pneumoniae* infections, with liver abscess cases constituting merely 25% of hmKP-infected patients. We detected hmKP strains in almost all specimen types and in patients receiving treatment in different departments, with a wide age range. A high proportion of ICU patients with hmKP infection and high levels of inflammatory indicators (WBC count and CRP and PCT levels) indicated the high pathogenicity of the isolated hmKP strains. Similar to hvKP, hmKP infection is also common in diabetes patients. In the present study, diabetes patients accounted for 39.8% of the patients with hmKP infection.

The phylogenetic tree showed that K1 and K64, K64 and K22, and K23 and K30 were from the same branch, indicating that these serotypes evolved from the same clone. Similarly, different sequence types on the same branch showed phylogenetic relationships, such as ST23 and ST5214, ST65 and ST375, and ST11 and ST34; moreover, some phylogenetic relationships were reported in the literature (Sanikhani et al., 2021; Michael et al., 2023; Jiang et al., 2024). Among the isolated hmKP strains, the capsular serotypes K1/K2 and sequence types ST23/ST65 were predominant, and their distribution was similar to those reported for hvKP, which indirectly indicated that a major proportion of the isolated hmKP strains was hvKP. Previous studies (Jin et al., 2021; Wang et al., 2023; García García B, Fernández-Manteca et al., 2024) have established correlations between specific capsule serotypes and sequence types, notably K1 with ST23/ST700 and K2 with ST65/ST25. Our findings not only confirm these associations but also reveal novel, strong pairings. The most striking is the K54/ST29 association (OR = 865.00), suggesting a previously under-characterized clone of potential importance. Other notable novel associations include K57/ST412 and K20/ST893. Although statistically significant (p < 0.05), the associations for K1/ST700 and K2/ST380 showed very wide confidence intervals (extending near 1), indicating low precision due to limited sample sizes. Therefore, these specific findings require validation in larger cohorts.

In July 2024, the World Health Organization issued a precautionary report stating the global spread and risk of hvKP infection. The hvKP strain, particularly ST23 type, can cause fatal infections even in immunocompetent populations due to its drug resistance property. In the present study, 76 hvKP strains were detected among the 88 hmKP isolates, accounting for 86.4% of the total isolates. Among these 76 hvKP strains, 28 strains were ST23 type hvKP, indicating that the ST23 type hvKP strain is highly prevalent in hmKP isolates. Moreover, 25% (n = 7) ST23 type hvKP strains were resistant to third-generation cephalosporins. In particular, a ST23 type HCKP strain was detected. Another ST23 type hvKP strain was sensitive to carbapenems but carried the *bla*_KPC-2_ resistance gene. These findings presented new challenges to combat this harmful pathogen. Because of the simultaneous occurrence of high virulence and antibiotic resistance, we expect that the risk of transmission of such strains will remarkably increase. The transmission or outbreak of such pathogenic strains in hospitals could lead to a disastrous outcome; hence, it is very critical to take necessary steps to prevent the spread of these highly pathogenic strains.

An important characteristic of *K. pneumoniae* is its ability to carry multiple virulence factors, including capsule, lipopolysaccharides, pili, siderophores, allantoin utilization, outer membrane proteins, and type 6 secretory system (Ombada et al., 2023; Park, 2023; Ragheb, 2024; Wang et al., 2024). Among these, capsule and siderophores are the primary virulence factors (Wang et al., 2020; Cardoso Almeida et al., 2024). The virulence genes *rmpA* and *rmpA2* regulate the synthesis of the extracellular polysaccharide capsule and are responsible for the hypermucoviscous phenotype (Kim et al., 2022; Yomogida D, Kuwano et al., 2024), as evidenced by their high prevalence in hmKP strains. *K. pneumoniae* expresses four types of siderophores: aerobactin, enterobactin, salmochelin, and yersiniabactin, with aerobactin being the most predominant one (Marr, 2019; Le et al., 2022). The relevant virulence determinants mainly include the *iuc*, *ent*, *iro*, and *ybt* genes encoding aerobactin, enterobactin, salmochelin, and yersiniabactin, respectively (Martin, 2018; Yang Y, Yang et al., 2021; Tanimoto et al., 2022). As shown previously, aerobactin is often concomitant with hypermucoviscosity (Choby et al., 2020), which was also validated in the present study. In this study, the isolated hmKP strains commonly carried the virulence determinants such as *rmpA*, *rmpA2*, *iuc*, *iro*, *peg-344*, and *clb*, indicating the strong virulence of these isolates. *mrk* is involved in the formation of pili and plays an important role in biofilm formation (Tada et al., 2023); strains carrying *mrk* can easily cause infections of indwelling catheters and drainage tubes (Russo et al., 2018). In the present study, *mrk* was detected in almost all *K. pneumoniae* strains, indicating that patients with indwelling catheters and drainage tubes should be cautious about *K. pneumoniae* infection development.

In the present study, although several hmKP strains were susceptible to the tested antibiotics, we should remain cautious about the emergence of multidrug-resistant (MDR) hmKP strains. Here, we detected 8 MDR hmKP strains, implying that these strains were resistant to three or more types of antibiotics. Especially the two cases of HCKP in this study, which pose a huge challenge for clinical diagnosis and treatment. Therefore, early identification, treatment, and prevention of the spread of such pathogens are highly critical steps to prevent future epidemics associated with these strains.

We also found that the detected resistance genes and antibiotic resistance were not completely consistent. The detection of resistance genes does not necessarily imply resistance to the specific antibiotic. Similarly, antibiotic resistance does not indicate the presence of resistance genes; this phenomenon is well explained in the results for quinolone and β-lactam resistance genes and antibiotic susceptibility. The *oqxA* and *oqxB* genes encoding quinolone resistance were present in almost all strains; however, the resistance rates to quinolone antibiotics (ciprofloxacin and levofloxacin) were relatively low and did not exceed 30%.

Although this study presents the epidemiological characteristics of hmKP infection, it has some limitations. First, this was a single-center study; therefore, the results may not be fully representative of the global scenario. Second, the sample size was relatively small, which may have resulted in biased findings. Nevertheless, the results of the present study contribute to the effective identification of hmKP infection and provide insights into the molecular characteristics of hmKP strains; these findings could aid clinical practice and facilitate the rapid and effective diagnosis of patients with hmKP infection.

## Conclusions

HmKP infection is prevalent in Yantai, China, with dominant K1/ST23 and K2/ST65 clones carrying prevalent virulence genes. Notably, HCKP strains were detected locally. Enhanced vigilance against such high-risk strains is urgently needed to guide local clinical response and infection control.

## Data Availability

The original contributions presented in the study are publicly available. This data can be found here: https://doi.org/10.6084/m9.figshare.31242130.
